# Supplementary material for: The Virome of Acute Respiratory Diseases in Individuals at Risk of Zoonotic Infections
Source: Viruses. 2020 Aug 29;12(9):960. doi: 10.3390/v12090960 (PMC7552073; doi:10.3390/v12090960)
Supplement: Supplementary file 1 [file viruses-12-00960-s001.pdf]

viruses

MDPI

Supplementary

The Virome of Acute Respiratory Diseases in Individuals at Risk of Zoonotic Infections

Tu Thi Kha Nguyen [1,2,3,*], Hong Thi Thu Nguyen [2], Ny Thi Han Nguyen [2], Phuc My Tran [2], Tam Thi Thanh Pham [2], H. Rogier van Doorn [4,5], Nghia Dang Trung Ho [2], Huong Thao Dang [2], Han An Duong [3], Ha Thi Thu Luu [3], Xutao Deng [6,7], Guy Thwaites [2,5], Eric Delwart [6,7,†], Anna-Maija K. Virtala [8,†], Olli Vapalahti [1,8,9,†], Stephen Baker [10,†] and Tan Van Le [2,*], on behalf of the VIZIONS consortium

[1] Doctoral School in Health Sciences, Faculty of Medicine, University of Helsinki, 00014 Helsinki, Finland; olli.vapalahti@helsinki.fi

[2] Oxford University Clinical Research Unit, Ho Chi Minh City 7000, Vietnam; hongntt@oucru.or (H.T.T.N.); nynth@oucru.org (N.T.H.N.); phuctm@oucru.org (P.M.T.); tamptt@oucru.org (T.T.T.P.); nghiahdt@oucru.org (N.D.T.H.); huongdt@oucru.org (H.T.D.); gthwaites@oucru.org (G.T.)

[3] Dong Thap Provincial Center for Disease Control, Dong Thap Province 660273, Vietnam; anhanduong@gmail.com (H.A.D.); luuthithuha2018@gmail.com (H.T.T.L.);

[4] Oxford University Clinical Research Unit, Ha Noi 8000, Vietnam; rvandoorn@oucru.org

[5] Centre for Tropical Medicine and Global Health, Nuffield Department of Medicine, University of Oxford, Oxford OX3 7LG, United Kingdom

[6] Department of Laboratory Medicine, University of California, San Francisco, CA, CA 94143, USA; XDeng@bloodsystems.org (X.D.); Eric.Delwart@ucsf.edu (E.D.)

[7] Vitalant Research Institute, San Francisco, CA 94118, USA

[8] Department of Veterinary Biosciences, Faculty of Veterinary Medicine, University of Helsinki, 00014 Helsinki, Finland; anna-maija.virtala@helsinki.fi

[9] Virology and Immunology, HUSLAB, Helsinki University Hospital, 00029 Helsinki, Finland

[10] Cambridge Institute of Therapeutic Immunology & Infectious Disease (CITIID), Department of Medicine, University of Cambridge, Cambridge CB2 0QQ, UK; sgb47@medschl.cam.ac.uk

* Correspondence: tuntk@oucru.org (T.T.K.N.); tanlv@oucru.org (T.V.L.); Tel: +84-89241761 (T.T.K.N.); Tel: +84-89241761 (T.V.L.)

† These authors contributed equally to this work.

Abstract: The ongoing coronavirus disease 2019 (COVID-19) pandemic emphasizes the need to actively study the virome of unexplained respiratory diseases. We performed viral metagenomic next-generation sequencing (mNGS) analysis of 91 nasal-throat swabs from individuals working with animals and with acute respiratory diseases. Fifteen virus RT-PCR-positive samples were included as controls, while the other 76 samples were RT-PCR negative for a wide panel of respiratory pathogens. Eukaryotic viruses detected by mNGS were then screened by PCR (using primers based on mNGS-derived contigs) in all samples to compare viral detection by mNGS versus PCR and assess the utility of mNGS in routine diagnostics. mNGS identified expected human rhinoviruses, enteroviruses, influenza A virus, coronavirus OC43, and respiratory syncytial virus (RSV) A in 13 of 15 (86.7%) positive control samples. Additionally, rotavirus, torque teno virus, human papillomavirus, human betaherpesvirus 7, cyclovirus, vientovirus, gemycircularvirus, and statovirus were identified through mNGS. Notably, complete genomes of novel cyclovirus, gemycircularvirus, and statovirus were genetically characterized. Using PCR screening, the novel cyclovirus was additionally detected in 5 and the novel gemycircularvirus in 12 of the remaining samples included for mNGS analysis. Our studies therefore provide pioneering data of the virome of acute-respiratory diseases from individuals at risk of zoonotic infections. The mNGS protocol/pipeline applied here is sensitive for the detection of a variety of viruses, including novel ones. More frequent detections of the novel viruses by PCR than by mNGS on the same samples suggests that PCR remains the most sensitive diagnostic test for viruses whose genomes

are known. The detection of novel viruses expands our understanding of the respiratory virome of animal-exposed humans and warrant further studies.

Keywords: virome; acute respiratory disease; NGS; metagenomics; zoonoses; novel cyclovirus; novel statovirus; novel gemycircularvirus

---

Table S1. Primer (and probe) sequences of the RT-PCRs used.

Primer-probe name	Sequence	Sources
Rotavirus-F	ACC ATC TWC ACR TRA CCC TC	
Rotavirus-R	GGT CAC ATA ACG CCC CTA TA	[1]
Rotavirus-Probe	FAM-ATG AGC ACA ATA GTT AAA AGC TAA CAC TGT CAA-BHQ1	
Vientovirus-inverse_F	TATTTGTGGCCTTACTCCTTGT	newly designed
Vientovirus-inverse_R	GGACATATAGCAGAAAAAGGTGATG	
CyCV-VZ13-confirm_F	ATCTCGCGCCCAATTTTGAAC	
CyCV-VZ13-confirm_R	ATGTCCACGCTCGGTTTGAC	
CyCV-VZ13-inverse_F	GTCGATGAATTAGAAACGTTTTGCA	newly designed
CyCV-VZ13-inverse_R	CTGGCATAACCCTGGAGGTG	
CyCV-VZ13-closing gap-F	AGAACCTTGTAGCGCGTGTT	
CyCV-VZ13-closing gap-R	GAGCGCCGCATAACGGTAAA	
GemyCV-VZ13-confirm_F	ATCAACCCGAACACGACGAA	
GemyCV-VZ13-confirm_R	CGGTGCCCAAGAAACCTTTG	
GemyCV-VZ13-inverse_F	TTCGTCGTGTTCGGGTTGAT	newly designed
GemyCV-VZ13-inverse_R	CAAAGGTTTCTTGGGCACCG	
GemyCV-VZ13-walking_F	GATGTCGAGCTCCGGGCATT	
GemyCV-VZ13-walking_R	CGCCATGTTGTAAGAAATCCACTC	
StatoV-VZ13-confirm_F	TGGAGCAAGCACATCATTGG	newly designed
StatoV-VZ13-confirm_R	GGCAGAAAGGTATCGGGTCA	
Gemycirculavirus-nested-outter-F	GTGGTAATGGTCGTCGGTATTC	
Gemycirculavirus-nested-outter-R	CCTCATCATTCGTAGTAAGCAATCTCA	[2]
Gemycirculavirus-nested-inner-F	AGTCCTGAATGTTTCCACTCG	
Gemycirculavirus-nested-inner-R	CAAGCGTTCCCTCGAAAATGAC	
Statovirus-F	GGYCTICTTGCWAARHTATG	[3]
Statovirus-R	RTCTKRYCCTCTRCATGGTC	

Table S2. Detail characteristics and virus detections of the samples at respiratory-disease episodes.

Sample ID	Cohort member ID	Date of disease episode (dd/mm/yy)	RT-PCR*	mNGS			mNGS-derived PCR[#]	
			Virus detected (Ct value)	No. of unique viral reads	Viral reads (%)	Virus** detected	Novel cyclovirus^	Novel gemycircularvirus^^
1	08-01	19-04-13	Negative	212	0.71			
2	20-02	11-05-13	Negative	970	1.60	Torque teno virus	detected by PCR	
3	08-01	23-05-13	Negative	1480	2.84			
4	81-23	05-06-13	Influenza A virus (29)	38	1.07	Influenza A virus (subtype N2)		
5	81-26	06-06-13	HRV (38)	874	2.34	HRV B3, human betaherpesvirus 7		
6	45-01	06-06-13	CoV[##] (36)	73	2.17	CoV subtype OC43		
7	47-01	06-06-13	Negative	142	2.07			
8	81-15	18-06-13	Negative	115	1.78			detected by PCR
9	81-16	18-06-13	Negative	180	2.12			
10	38-01	20-06-13	Negative	277	1.61			detected by PCR
11	38-02	20-06-13	Negative	243	1.66	Statovirus A3		
12	38-04	20-06-13	Negative	52	1.63			
13	43-03	20-06-13	Negative	62	1.54			
14	43-04	20-06-13	Negative	45	0.52			
15	49-01	20-06-13	Negative	1143	3.14	Human papillomavirus		
16	51-02	20-06-13	Negative	282	1.03			detected by PCR
17	81-23	10-07-13	Negative	304	0.98	Human papillomavirus		detected by PCR
18	48-01	10-07-13	Negative	566	1.93			
19	50-01	10-07-13	Negative	817	1.75			
20	60-01	09-07-13	Negative	270	1.41			
21	60-02	14-07-13	Negative	263	1.91			
22	60-03	09-07-13	Negative	11,506	2.40	Novel cyclovirus^	*detected by mNGS*	
23	60-04	09-07-13	Negative	555	2.68	Novel statovirus^^^, MPV		
24	60-07	09-07-13	Negative	3446	3.16			

25	10-01	19-07-13	Negative	224	1.03			
26	08-01	24-07-13	Negative	20,665	5.43			
27	36-01	30-07-13	Negative	42	0.66			detected by PCR
28	53-01	30-07-13	Negative	391	1.71			
29	53-03	30-07-13	Negative	150	0.48			detected by PCR
30	22-01	08-08-13	Negative	2720	1.61	Novel gemycircularvirus^^, novel statovirus^^^		*detected by mNGS*
31	21-03	08-08-13	HRV (40)	13,498	3.74	EVs D68		
32	81-17	22-08-13	Negative	1472	1.73			
33	60-01	09-09-13	Negative	900	2.12			
34	60-01	20-09-13	Negative	284	2.62			
35	60-06	11-09-13	Negative	769	2.07			
36	60-06	20-09-13	Negative	697	2.03			
37	60-07	11-09-13	RSV A (38.6)	420	2.04			
38	60-07	21-09-13	Negative	546	1.80			
39	60-05	09-09-13	Negative	554	2.14		detected by PCR	
40	60-13	20-09-13	Negative	217	0.46			
41	61-01	05-09-13	Negative	1062	2.04			
42	81-20	11-09-13	Negative	192	2.09			
43	60-02	09-09-13	Negative	2401	2.52			
44	60-11	19-09-13	Negative	881	1.99			
45	60-10	19-09-13	Negative	338	1.20	RSV A		
46	60-03	20-09-13	Negative	520	2.92			
47	43-02	30-09-13	Negative	162	1.18			
48	43-03	30-09-13	Negative	133	2.14			
49	43-04	30-09-13	Negative	159	1.89			
50	60-05	16-10-13	RSV A (31)	1003	1.37	RSV A genotype ON1	detected by PCR	
51	61-04	16-10-13	Negative	114	1.23			
52	61-02	18-10-13	HRV (40)	181	2.15	HRV B		
53	61-05	18-10-13	Negative	109	0.72			
54	18-02	22-10-13	Negative	1269	0.48			
55	61-10	24-10-13	Negative	2703	1.73			detected by PCR
56	81-13	24-10-13	Negative	131	1.80			

57	81-09	28-10-13	Negative	312	1.62			
58	61-13	28-10-13	Negative	440	1.47			detected by PCR
59	61-01	28-10-13	Negative	909	1.71			
60	81-10	28-10-13	Negative	346	1.40			detected by PCR
61	81-08	31-10-13	Negative	1236	2.05			
62	61-05	08-11-13	Negative	529	2.33			
63	05-03	08-11-13	MPV (40)	850	1.89			
64	61-13	11-11-13	Negative	249	1.16			detected by PCR
65	60-07	15-11-13	Negative	1076	1.47			
66	60-13	15-11-13	Negative	12352	4.06			
67	60-12	19-11-13	Negative	566	2.41			
68	61-05	25-11-13	Negative	1551	2.27	Torque teno virus		
69	61-04	25-11-13	Negative	1534	1.40	Gemycircularvirus, statovirus		
70	60-13	26-11-13	HRV (37.1), EVs (32.4)	53595	12.69	Coxsackievirus A21, HRV C56		
71	63-02	29-11-13	HRV (40)	7582	3.67	HRV B86, vientovirus		
72	63-01	29-11-13	Negative	414	0.56			
73	60-13	17-12-13	EVs (39)	1002	1.98	HRV B		
74	60-06	18-12-13	Negative	688	1.68		detected by PCR	
75	60-03	18-12-13	Negative	290	1.73		detected by PCR	
76	60-01	18-12-13	Negative	45	1.64			
77	60-07	18-12-13	Negative	713	1.20			
78	81-11	23-12-13	Negative	637	1.24			detected by PCR
79	81-14	23-12-13	Negative	11546	5.30			
80	60-12	24-12-13	Negative	3075	3.65	Novel statovirus^^^		detected by PCR
81	60-11	26-12-13	HRV (39)	11026	4.35	HRV B79, novel gemycircularvirus^^		*detected by mNGS*
82	60-08	26-12-13	Negative	439	1.25			
83	45-01	26-12-13	Negative	318	2.88	Circular virus of Circoviridae family		
84	43-02	26-12-13	HRV (38)	20038	6.24	HRV B79		
85	43-03	26-12-13	Negative	263	2.38	Gemycircularvirus		
86	43-04	26-12-13	Negative	416	2.45			

87	47-01	26-12-13	Negative	1124	2.42	Rotavirus, novel gemycircularvirus^^	*detected by mNGS*
88	14-03	30-12-13	Negative	231	1.87		
89	17-01	30-12-13	HRV (40)	2782	2.05	HRV A57	
90	17-03	30-12-13	HRV (37)	13941	5.10	HRV B35, bat badicivirus, bat posalivirus	
91	25-02	30-12-13	Negative	5952	5.41		
NC	Negative control: viral transport medium			19	0.27		

* done and reported previously [4].

** viruses that have previously been detected in human samples.

PCR screening (confirmed by Sanger sequencing) with new primers designed based on mNGS contigs.

subtype OC43 and/or NL63.

^ provisionally named CyCV-VZ13.

^^ provisionally named GemyCV-VZ13.

^^^ provisionally named StatoV-VZ1

Table S3. Mix-detection of viruses detected by NGS with PCR confirmation.

NTS ID	Co-infections	Time point of collection
1	CyCV-VZ13 and GemyCV-VZ13	Baseline sample
2	CyCV-VZ13 and GemyCV-VZ13	Baseline sample
3	CyCV-VZ13 and GemyCV-VZ13	Baseline sample
4	CyCV-VZ13 and Statovirus-VZ13	Disease episode
5	CyCV-VZ13 and RSVA	Disease episode
6	GemyCV-VZ13 and Statovirus-VZ13	Disease episode
7	GemyCV-VZ13 and Statovirus-VZ13	Disease episode
8	GemyCV-VZ13 and HRV B79	Disease episode
9	GemyCV-VZ13 and Rotavirus	Disease episode

Table S4. Detection of viruses of invertebrate, insect, plant, fungi, bacteria and algae.

	Family	Species
**Invertebrate viruses**	*Iridoviridae*	Orange-spotted grouper iridovirus
	Dicistroviridae	Drosophila C virus
**Insect viruses**	*Baculoviridae*	*Galleria mellonella* MNPV
	Dicistroviridae	Drosophila C virus
	Iflaviridae	Diamondback moth iflavirus
	Polydnaviridae	Proviruses
**Plant viruses**	*Betaflexiviridae*	Shallot latent virus, Garlic latent virus,
	Bromoviridae	Cucumber mosaic virus
	Potyviridae	Chilli veinal mottle virus
	Partitiviridae	Fig cryptic virus
**Fungal viruses**	*Chrysoviridae*	Chrysoviruses
	Totiviridae	Totiviruses
**Bacterial viruses (phages)**	*Siphoviridae*	*Streptococcus* viruses, *Arthrobacter* phages, Clostridium phages, *Lactobacillus* phages, *Microbacterium* phages
	Inoviridae	*Vibrio* phages
	Myoviridae	*Acinetobacter* phages, *Aeromonas* phages, *Bacillus* phages, *Campylobacter* viruses, *Corynebacterium* phages, *Klebsiella* phages
	Podoviridae	*Actinomyces* viruses, *Lactococcus* viruses
	Leviviridae	Leviviruses
**Algae viruses**	*Phycodnaviridae*	*Chrysochromulina ericina* viruses

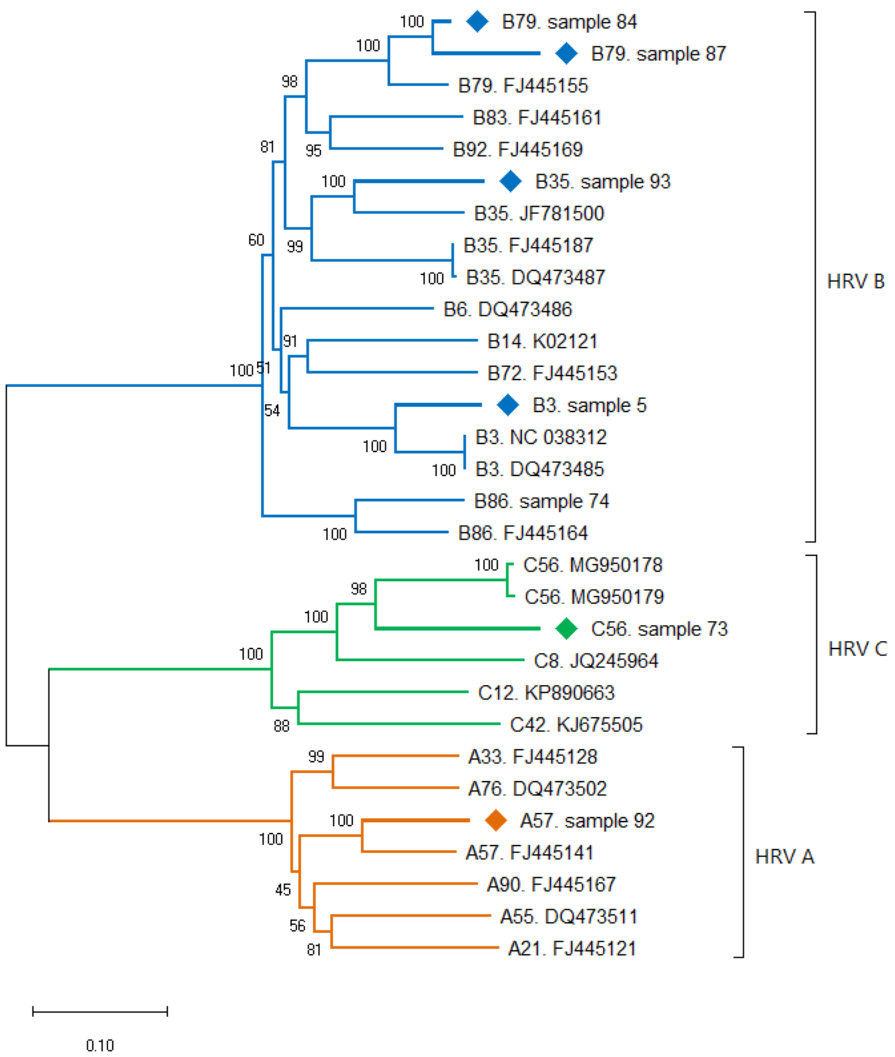

Figure S1. Phylogenetic tree of partial VP1-VP3 protein sequences (90% coverage) from HRV sequences obtained from mNGS herein against corresponding sequences from The US on GenBank.

References

1. Dung, T.T.N.; Phat, V.V.; Nga, T.V.T.; My, P.V.T.; Duy, P.T.; Campbell, J.I.; Thuy, C.T.; Hoang, N.V.M.; Minh, P.V.; Phuc, P.L.; et al. The validation and utility of a quantitative one-step multiplex RT real-time PCR targeting rotavirus A and norovirus. *J. Virol. Methods*. **2013**, *187*, 138–143, doi:10.1016/j.jviromet.2012.09.021
2. Anh, T.N.; Hong, T.T.N.; Nhu, N.L.T.; Thanh, T.T.; Lau, C.-Y.; Limmathurotsakul, D.; Deng, X.; Rahman, M.; Chau, V.V.N.; van Doorn, H.R.; et al. Viruses in Vietnamese patients presenting with community acquired sepsis of unknown cause. *J. Clin. Microbiol.*, **2019**, *57*, e00386–19, doi: 10.1128/JCM.00386-19.
3. Janowski, A.B.; Krishnamurthy, S.R.; Lim, E.S.; Zhao, G.; Brenchley, J.M.; Barouch, J.H.; Thakwalakwa, C.; Manary, M.J.; Holtzet, L.R.; et al. Statoviruses, A novel taxon of RNA viruses present in the gastrointestinal tracts of diverse mammals. *Virology* **2017**, *504*, 36–44, doi:10.1016/j.virol.2017.01.010
4. Nguyen, T.T.K; Ngo, T.T.; Tran, P.M.; Pham, T.T.T.; Vu, H.T.T; Nguyen, N.T.H.; Thwaites, G.; Virtala, A.-M.K.; Vapalahti, O.; Baker, S.; et al. Respiratory viruses in individuals with high frequency of animal exposure in southern and highland Vietnam. *J. Med.l Virol*. **2019**, doi:10.1002/jmv.25640
